# CAR T-cells meet autoimmune neurological diseases: a new dawn for therapy

**DOI:** 10.3389/fimmu.2025.1604174

**Published:** 2025-07-18

**Authors:** Nikolaos A. Chinas, Harry Alexopoulos

**Affiliations:** Department of Cell Biology and Biophysics, Faculty of Biology, National and Kapodistrian University of Athens, Athens, Greece

**Keywords:** autoimmune neurological diseases, CAR T-cells, preclinical data, clinical trials, CRS, efficacy, safety, limitations

## Abstract

Autoimmunity and autoimmune diseases arise when the immune system erroneously targets self-antigens leading to tissue damage. Consequently, immunomodulatory and mainly immunosuppressive drugs comprise the conventional treatment in conditions such as systemic lupus erythematosus, rheumatoid arthritis and multiple sclerosis. However, many of these agents often fall short of providing a cure and have a limit on symptom management. This underscores the urgent need for even more advanced therapies for patients to constrain progressive disability. Therefore, currently, researchers explore the potential of chimeric antigen receptor (CAR) T-cell therapy for autoimmune diseases considering its success in cancer treatment and specifically in hematological malignancies. This review will examine recent advancements in CAR T-cell therapy for autoimmune disorders, highlighting how CAR T cells can be engineered to precisely target and eliminate autoreactive immune cells that drive these debilitating diseases, particularly those affecting the nervous system such as Multiple sclerosis, Myasthenia gravis, Neuromyelitis optica, Stiff-person syndrome, Autoimmune encephalitis, MOG-antibody disease and Chronic inflammatory demyelinating polyneuropathy. Also, through an analysis of preclinical and clinical data, we will assess the efficacy, safety, potential side effects and limitations of these innovative therapies. Lastly, we will underline the transformative potential of CAR T-cells in Autoimmune Neurology, offering a promising new hope for treatment where conventional therapies have failed.

## 1 Introduction

**Figure 1:**
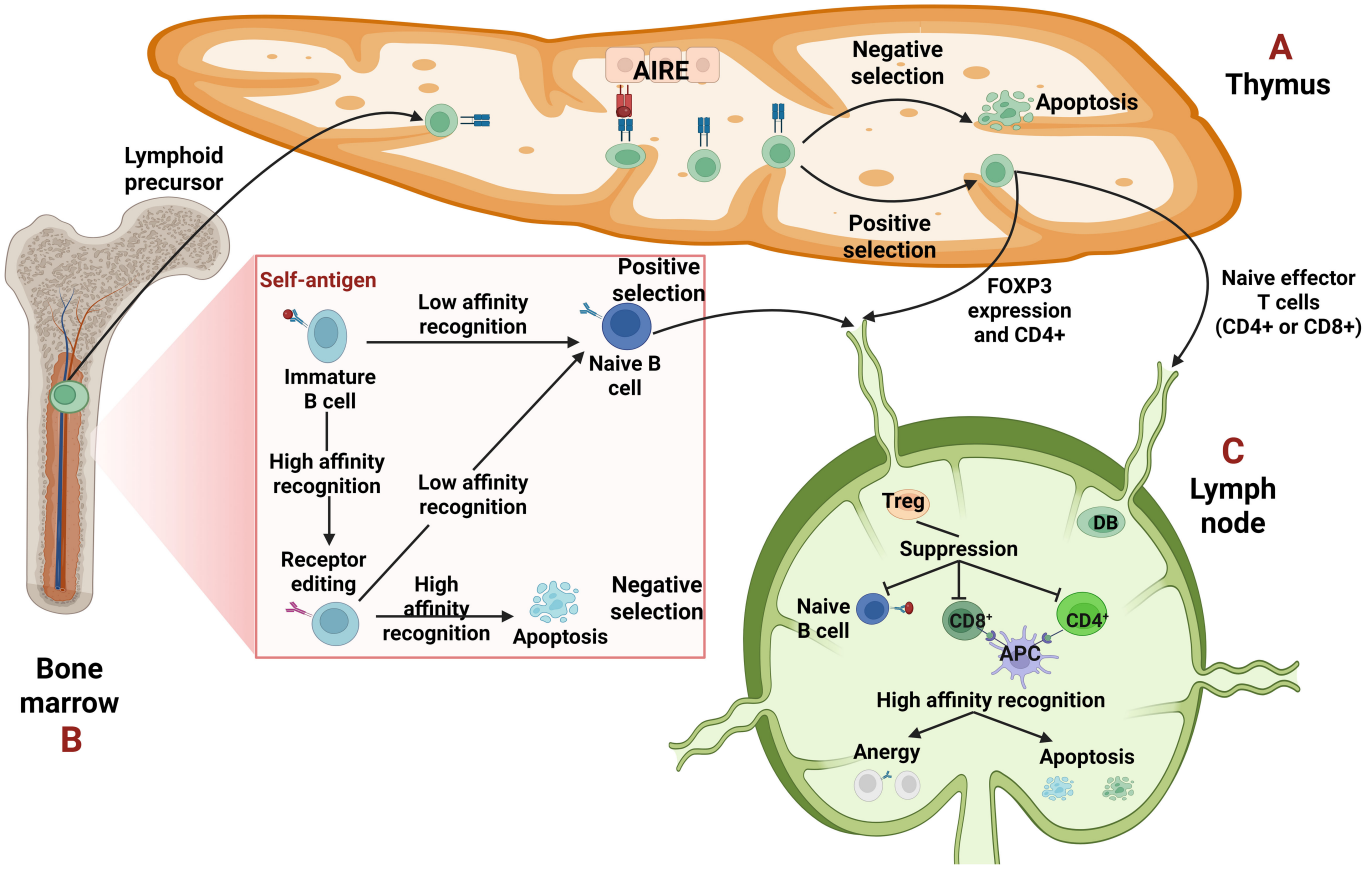
Central and peripheral tolerance. **(A, B)** Central tolerance takes place in the primary lymphoid organs, thymus for T cells and bone marrow for B cells. **(A)** The epithelial cells of the thymus, due to the transcription factor autoimmune regulator (AIRE) can express a wide variety of tissue specific antigens (TSAs) to promote self-tolerance. The T cells undergo selection, either negative, when their T cell receptor (TCR) recognizes strongly the self-antigen resulting in their apoptosis or positive, when the recognition is low. They leave the thymus as single positive (CD4+ or CD8+) T cells. Some T cells that recognize self-antigens with moderate affinity become T regulatory cells (Tregs) expressing forkhead box P3 (FOXP3) and CD4. **(B)** Regarding B cells, when their B-cell receptor (BCR) recognizes a self-antigen with high affinity, they edit their receptor through recombination of the light chain. If the edited receptor still possesses high affinity, the B cells will be led to negative selection or apoptosis. In case of a moderate affinity BCR, the B cells undergo positive selection and leave the bone marrow as naïve B cells. **(C)** Peripheral tolerance is orchestrated on peripheral tissues like lymph nodes, to hamper either already autoreactive lymphocytes that “slipped” from central tolerance or possible future autoreactive cells which will merge after they encounter a self-antigen. In periphery, autoreactive cells if they recognize a self-antigen without receiving co-stimulation, can become anergic -which means inactivated- or can be deleted through apoptosis. Tregs can suppress them through different mechanisms, too.

The immune system, though it is designed to defend us by eliminating pathogens, in cases where genetic and environmental factors conspire, directs its action on the host (1). Each healthy individual acquires a unique and diverse repertoire of T cell (TCR) and B cell receptors (BCRs) generated through somatic recombination in different genetic regions. These rearrangements can give rise to TCRs or BCRs that recognize not only a wide range of foreign antigens but also self-peptides (2, 3). Therefore, two main mechanisms ensure the elimination of autoreactive cells (3) as shown in **Figure 1**.

The first one is central tolerance where the apoptosis of T and B cells, with high affinity receptors -TCRs and BCRs- takes place early in their maturation in thymus and bone marrow, respectively (3). Specifically, T cells must undergo two selections during their maturation in the thymus: positive selection, in which a T cell must recognize a self-peptide presented by an MHC molecule and negative selection in which T cells that recognize self-peptides with high-affinity are deleted. Medullary thymic epithelial cells (mTECs) contribute to this process by expressing a variety of tissue specific antigens (TSAs) driven by the transcriptional activator autoimmune regulator (AIRE), thereby promoting self-tolerance through the “training” of T cells (3, 4). However, a subset of CD4+ T cells with intermediate affinity TCRs (5) develop into T regulatory cells (Tregs) through the upregulation of forkhead box P3 (FOXP3). The primary role of Tregs is to suppress autoreactive cells in peripheral tissues (3–5). On the other hand, in the bone marrow, immature B cells must undergo a similar form of “training” to ensure self-tolerance (3, 6). When an immature B cell recognizes a self-antigen with high affinity, it is given a second chance to alter its specificity reactivating the recombination machinery through a process known as receptor editing (2, 3, 6). If receptor editing is successful and the newly generated BCR is non-autoreactive, the B cell undergoes positive selection and is permitted to migrate to the periphery. Conversely, if receptor editing fails and the BCR remains autoreactive, the B cell is eliminated through negative selection and apoptosis (3, 6).

The second mechanism that ensures the elimination of autoreactive cells is peripheral tolerance, which acts as a backup when central tolerance fails to eliminate all self-reactive cells (3). Autoreactive cells that escape central tolerance may reach secondary lymphoid organs, where they can become anergic (inactivated) or apoptotic (3–5). Anergy is a state of functional unresponsiveness due to the lack of co-stimulation (3, 7). For example, the activation of T cells requires two signals: the first one is the recognition of a pMHC presented by an antigen-presenting cell (APC) and the second is the interaction of co-stimulatory receptors −specifically, CD28 on the T cell and B7 molecules (CD80/CD86) on the APC. In the absence of this second signal, the T cell becomes anergic (7). Tregs play a major role in the suppression of autoreactive cells through different ways. First, they cause metabolic disruption by consuming survival and proliferation cytokines such as interleukin-2 (IL-2) or B-cell Activating Factor (BAFF). They also express inhibitory receptors, notably Cytotoxic T Lymphocyte-Associated Protein 4 (CTLA-4), which competes with CD28 for binding to B7. Lastly, Tregs secrete inhibitory cytokines such as IL-10, IL-35 and TGF-β (3–6).

In autoimmunity, the above mechanisms are partly incapacitated (3, 4) giving rise to over 80 autoimmune diseases (8).

Many of these autoimmune diseases affect the nervous system directly or indirectly. Those which target the nervous system *per se* are called neuro-autoimmune disorders. In these disorders, autoreactive cells, either through the production of auto-antibodies (Abs) or directly through their receptors target self-antigens of the central (CNS) or peripheral (PNS) nervous system causing damage to cells and organs. Multiple sclerosis (MS) and Chronic Inflammatory Demyelinating Polyneuropathy (CIDP) are key examples of CNS and PNS autoimmune diseases, respectively. These diseases can lead to significant neurological impairments (9). There are cases though, where CNS and PNS pathology occur simultaneously (10).

Autoimmune diseases while exhibiting different pathologies have the same culprits; autoreactive B and T-cells initiate the attack on autoantigen-expressing tissues directly through their Abs or TCRs, respectively or through cytokine production which mobilizes innate immunity (11, 12). As a result, the main therapeutic options are centered on the prevention of these immune cells from perpetuating tissue damage and this can be achieved with immunosuppressive and immunomodulatory agents such as glucocorticoids, and a variety of monoclonal antibodies (mAbs). For example, Rituximab is a mAb which targets CD20+ B-cells and leads to B-cell depletion in MS patients, while TNF-α (tumor necrosis factor-α) inhibitor, Infliximab, is used in Rheumatoid Arthritis (RA) and Crohn’s disease (12). Especially for MS treatment, FDA, in the last two decades, has approved newer classes of drugs, all under the umbrella of disease-modifying therapies (DMTs) for the control of disease progression (13).

However, some conventional therapies make patients susceptible to infections due to severe immunosuppression (i.e. cyclosporin, dexamethasone) while a significant proportion of them respond poorly (12, 14). Also, the need for repeated administration and the high cost and effort to engineer humanized mAbs to alleviate undesirable immune responses made scientists pursue new therapies (12, 15).

The success of CAR T-cell therapy in the treatment of B-cell hematologic malignancies led researchers to investigate the feasibility of using CAR T-cells in autoimmune diseases which are mediated by autoreactive B-cells such as MS, Systemic Lupus Erythematosus (SLE) and Pemphigus Vulgaris (PV) (16–21). To understand the extent of this success, patients with Diffuse Large B-Cell Lymphoma (DLBCL) and Relapsed/Refractory Acute Lymphoblastic Leukemia (R/R ALL), when treated with Tisagenlecleucel, an FDA-approved CAR T-cell product for CD19+ B-cells, achieved an objective response rate (ORR) of 52% and 81%, respectively (18, 19). There are also cases with progression-free survival of greater than 5 years, making CAR T-cell therapy one of the most successful treatments in cancer immunotherapy (20). Currently, there are five generations of CAR T-cells, but the six FDA-approved CAR T-cell therapies, which target ten hematological cancers, all belong to the second generation (21). One of the reasons for their success is the high selectivity for tumor cells and the minimization of the severe side effects that come with non-specific treatments like chemotherapy and radiation therapy (22). Consequently, research turned to CAR T-cell therapy for autoimmune diseases (23–27).

In this review, we attempt to expose the need for CAR T-cell therapy beyond cancer, evaluate the “cost-benefit” based on clinical studies and shed light on the limitations of CAR T-cell therapy in the treatment of autoimmune neurological diseases.

## 2 CAR T-cells

### 2.1 Manufacturing process and structure

**Figure 2:**
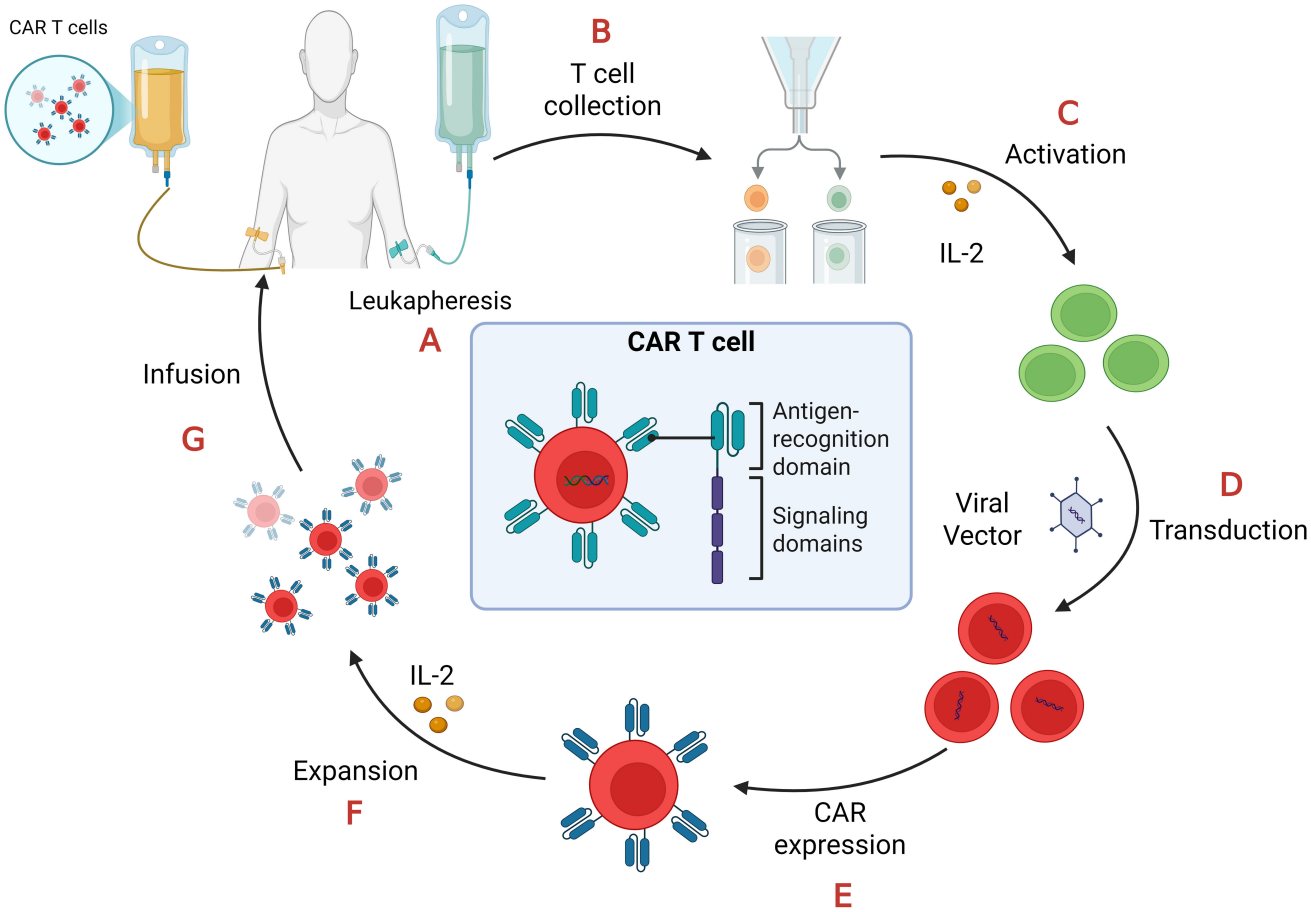
CAR-T cell manufacturing process. **(A)** Leukapheresis: collection of peripheral blood mononuclear cells (PBMCs); **(B)** T cell collection: separation of T cells from PBMCs.; **(C)** Activation: T cells require stimulation from artificial antigen-presenting cells (aAPCs), anti-CD3/anti-CD28 antibodies, and interleukin-2 (IL-2) in order to proliferate and become effector T cells; **(D)** Transduction: T cells are transduced with lenti- or retroviral vectors to express CAR on their cell membrane; **(E)** CAR expression: the viral vectors integrate the CAR gene in the T-cell genome so they express it on their surface; **(F)** Expansion: CAR-T cells proliferate after their treatment with IL-2; **(G)** Infusion: CAR-T cells, after quality and functional assessments, transferred back to the patient through intravenous infusion.

Beyond doubt the manufacturing of CAR T-cells is a tedious and expensive process (23, 27, 28) as depicted in **Figure 2**.

Prototype CARs are composed of four parts: an extracellular antigen binding domain, often consisting of a single-chain variable fragment (scFv), an extracellular spacer or hinge, a transmembrane domain, and an intracellular T cell activation component (29).

T-cells’ activation is dependent on the TCR recognition of antigens presented on Major Histocompatibility Complex (MHC) molecules by antigen-presenting cells. However, tumor cells evade the immune system through MHC downregulation or even loss (30). Therefore, CAR T-cells’ activation must not depend on MHC molecules, and this is accomplished with the single-chain variable fragment (scFv) of an antibody as the extracellular antigen binding domain. scFv consists of the variable heavy (VH) and light (VL) chains responsible for antigen binding. The two chains are connected by a flexible peptide linker which enhances the affinity of the receptor. scFv can recognize a wide range of antigens (proteins, lipids, carbohydrates or combination of them) that naturally generated TCRs cannot (28, 31). Also, the affinity of recognition plays a major role (32–34). High-affinity does not guarantee better antitumor responses. The moderate recognition (32) and increased hydrophobicity (33) enhance antigen-binding affinity optimizing scFv (32, 33). Thus, the selection of the antigen must be meticulously made (28, 32–34).

The hinge or extracellular spacer provides scFv with flexibility and connects it to the transmembrane domain (28, 34). Fc constant regions of IgG and IgD immunoglobulins or CD8 domains generally serve as the hinge (34). The extracellular domain must be able to bind to its target and that requires specific alignment between receptor-antigen. The specific geometry they must follow correlates with the efficacy of CAR T-cells, and it is important to select a specific hinge structure that will facilitate the proper binding and mitigate undesirable immune responses either by the CAR T-cells or the innate immune system (35, 36). Systematic testing of 360 CAR constructs suggests that the binding-affinity of the scFv is not affected only from the size of the hinge but also from the different scFv-hinge combinations. The hinge also plays a major role in the dynamics of the CAR T-cell activation due to the conformational changes it induces in the CAR receptor (37).

The transmembrane domain (TMD) it is the same as in naturally occurring immune receptors like CD3ζ (ζ-chain of the TCR complex) (28, 34). It stabilizes the CAR and anchors it in the cell membrane playing a role in signaling, too (28, 34, 36). Also, Müller et al. revealed that the CD28 as a TMD can form heterodimers with endogenous CD28 when either CD28 or CD8 serves as a hinge. These heterodimeric interactions can influence the functionality of CAR T cells in terms of cytokine production (38). Consequently, the pairing of the TMD and hinge region must be carefully considered during CAR design to ensure optimal therapeutic performance (36, 38).

**Figure 3:**
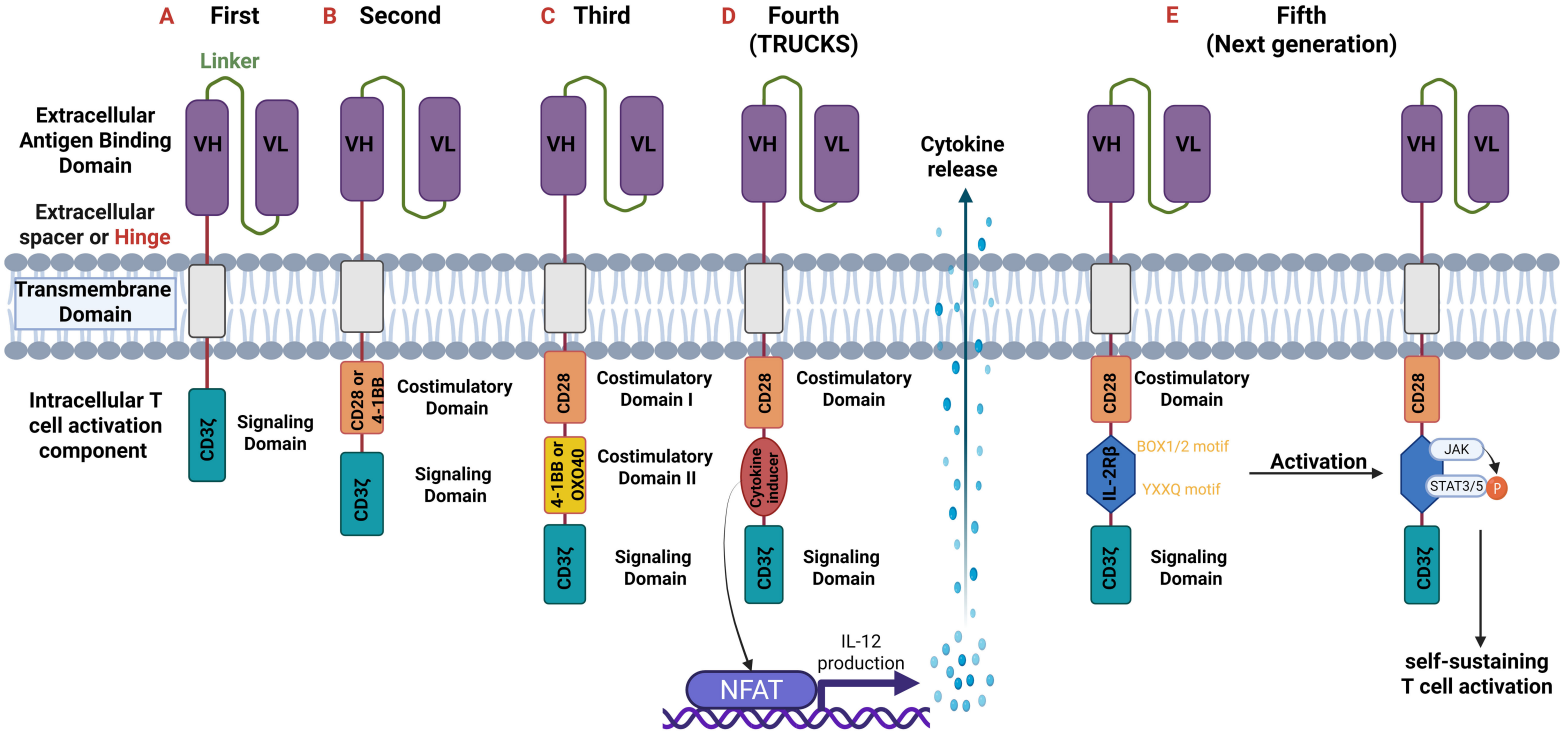
Generations of CARs. All generations have an extracellular antigen binding domain consisting of variable heavy (VH) and light (VL) chains (scFv). They differ though in the intracellular component. **(A)** First-generation: one intracellular CD3ζ for signal transduction.; **(B)** Second-generation: two intracellular domains: CD3ζ and an additional costimulatory domain (e.g., CD28 or 4-1BB).; **(C)** Third-generation: three intracellular domains: CD3ζ and two additional costimulatory domains (CD28 and 4-1BB or OXO40).; **(D)** Fourth-generation or T-cells redirected for universal cytokine-mediated killing (TRUCKS): are based on the second generation but with the addition of a cytokine inducer domain which activates the nuclear factor of activated T cells (NFAT). NFAT as a transcription factor induces cytokine production (especially of IL-12) which is secreted from CAR T-cells and thus modulate immune responses.; **(E)** Fifth or Next generation: based on the second generation but with the addition of intracellular domains of cytokine receptors such as IL-2 receptor β-chain (IL-2Rβ) domain with a binding site for Janus kinase (JAK) and for the transcription factors Signal transducer and activator of transcription 3/5 (STAT3/5). Antigen activation triggers three synergistic signals through CD3ζ, CD28, and cytokine JAK–STAT3/5 signaling, which lead to T-cell activation and proliferation. CD, cluster of differentiation.

The intracellular T-cell activation component is responsible for activating the CAR-T cell upon antigen binding. First generation CARs contain one intracellular signal domain, only the CD3ζ (28, 34, 39). Second and third generation ones have one and two extra costimulatory domains, respectively. These domains are 4-1BB (CD137), CD28, ICOS or OX40 (CD134). The fourth generation possesses a costimulatory domain and a protein production inducer (i.e. IL-2) which in the fifth generation is substituted with intracellular domains of cytokine receptors, such as IL-2 receptor β-chain (IL-2Rβ) fragment (39, 40). The structure of the five generations of CARs is demonstrated in **Figure 3**.

### 2.2 Main approaches of CARs for autoimmune diseases

Nowadays, there are two main approaches concerning CAR T-cell therapy for autoimmune diseases (23, 26, 27, 41, 42).

**Figure 4:**
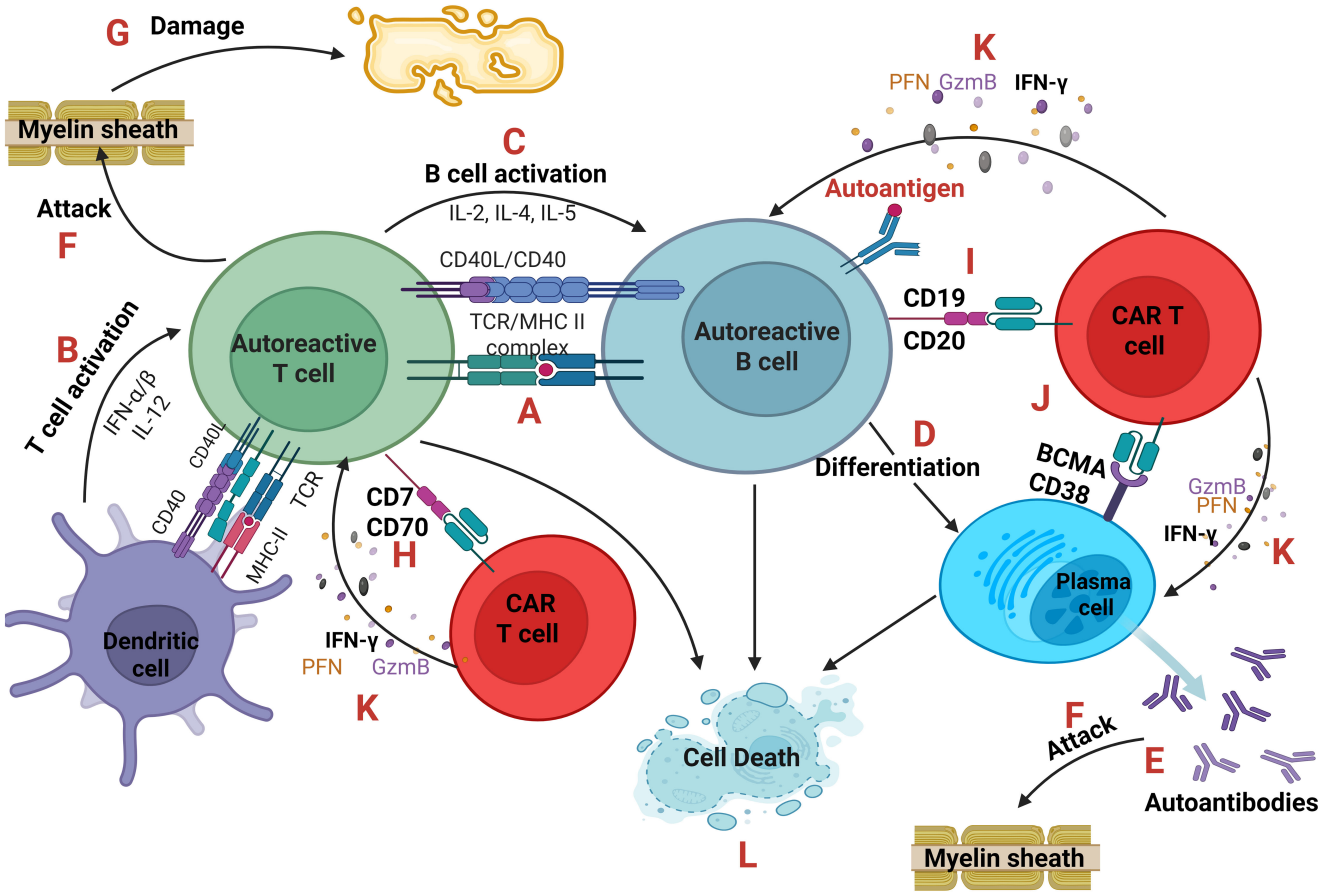
Autoreactive cells and CAR T-cells. An antigen-presenting cell (dendritic cell or autoreactive B cell) presents through the MHC class II a self-antigen to an autoreactive T cell **(A)** which becomes activated after the co-stimulation of CD40-CD40L and the secretion of cytokines IFN-α, IFN-β and IL-12. **(B)** In parallel, B cell activation is based on the secretion of IL-2, IL-4, IL-5 from T cells. **(C)** B cells after activation can differentiate into plasma cells **(D)** producing large quantities of autoantibodies **(E)** which along with autoreactive TCRs of T cells can directly attack the cells or tissues expressing the self-antigen like the Myelin Basic Protein (MBP) of the myelin sheath of oligodendrocytes which is believed to be the autoantigen in Multiple Sclerosis. **(F)** As a result, tissue damage occurs. **(G)** CAR T-cells can eliminate autoreactive T, B and plasma cells targeting CD70 or CD7, **(H)** CD19 or CD20 **(I)** and BCMA or CD38, **(J)** respectively. CAR T-cells through the secretion of Perforin (PFN), Granzyme B (GzmB) and IFN-γ **(K)** cause the cell death of autoreactive cells **(L)**. IFN, interferon; CD, cluster of differentiation.

The first one, as shown in **Figure 4**, emphasizes on the elimination of autoreactive cells by engineering CAR T-cells that will selectively kill autoreactive cells (41, 42).

**Figure 5:**
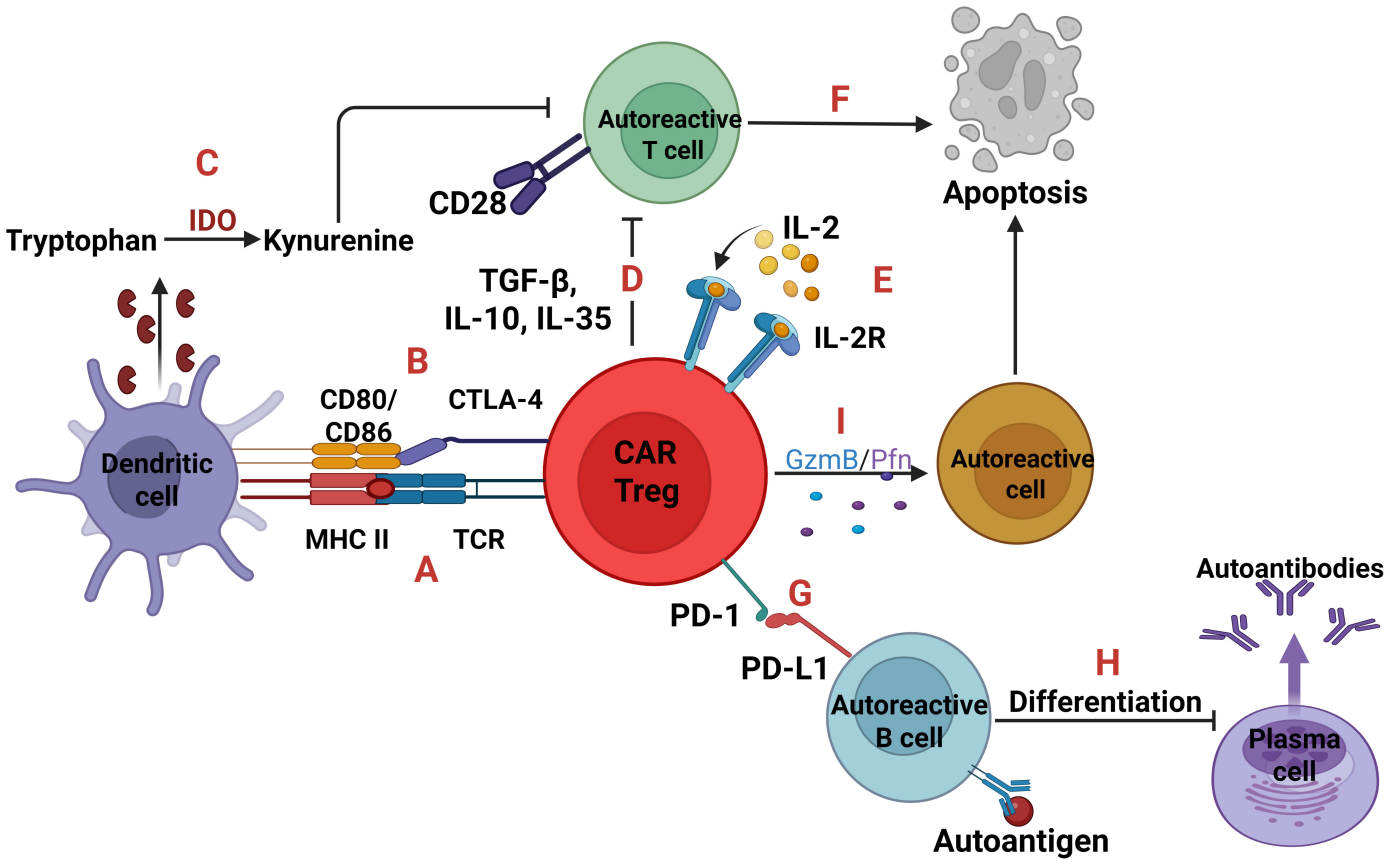
Autoreactive cells and CAR Tregs. Two signals are essential for T-cell activation; one is TCR-MHC signaling and the other is CD28-CD80/86 co-stimulation. CAR Tregs can express a TCR which binds to the complex MHC class II-autoantigen of dendritic cells leading to CAR Tregs proliferation **(A)**. Also, through the expression of Cytotoxic T-lymphocyte associated protein 4 (CTLA-4) which binds to the CD80/86 can inhibit the activation and maturation of dendritic cells, so as not to present the self-antigens to T cells **(B)**. In parallel, the CTLA-4 inhibition upregulates the Indoleamine 2, 3-dioxygenase (IDO) expression, an enzyme which converts tryptophan to kynurenine, with the later suppressing T cells **(C)**. Moreover, CAR Tregs either with the immunosuppressive cytokines TGF-β, IL-10, IL-35 **(D)** or with IL-2 deprivation **(E)** can cause T cell apoptosis **(F)**. Regarding autoreactive B cells, CAR Tregs expressing the receptor programmed cell death protein 1 (PD-1) which binds to programmed death-ligand 1 (PD-L1) **(G)**, can induce B cell tolerance, inhibiting their activation and differentiation into plasma cells **(H)**. Lastly, through the secretion of Granzyme B and Perforin trigger apoptosis of any autoreactive cell **(I)**.

The second approach, as illustrated in **Figure 5**, is based on the restoration of immunoregulation as it is disrupted in autoimmune disorders. The main concept is the engineering of CAR regulatory T-cells (Tregs) that will modulate the immune system (41–43). In 2023, researchers reported successful TCR-like CAR Tregs for type 1 diabetes (T1D) as its pathology is linked to compromised Tregs. Specifically, engineered CAR Tregs had a scFv mimicking TCR specificity for insulin epitopes presented by APCs. This approach prevented spontaneous T1D in mice (44, 45). CAR Tregs also protected grafted mice with stem cell-derived β cells which produce insulin from autoimmune attack (46).

CAR T-cells must target specific markers on B and T-cells that will be present from the pro-B and pro-T cells in the primary lymphoid organs (bone marrow and thymus, respectively) up to the mature-effector B and T-cells in the periphery. For this purpose, CD19, CD20, CD38, BCMA (B cell maturation antigen) and CD7, CD70, may serve as CAR-targets on B and T-cells, respectively (23, 41–43).

Another approach was introduced in 2016 by Ellebrecht et al. The researchers engineered CAAR T cells for pemphigus vulgaris (PV), an autoimmune skin condition where autoreactive B cells target desmoglein 3 (Dsg3). Researchers, instead of conventional CAR they expressed an epitope of Dsg3 on the cell surface of CAAR T cells. This approach allowed T cells to recognize and eliminate B cells that bear anti-Dsg3 BCRs (47).

## 3 Targeted autoimmune neurological diseases

Nowadays, as shown in **Table 1**, there are several ongoing clinical trials evaluating the safety, efficacy and dosage of CAR T-cells in autoimmune neurological diseases both of CNS and PNS.

**Table 1:** Ongoing clinical trials of CAR T-cells for the treatment of autoimmune neurological diseases.

Title	Participants (Estimated Enrollment)	Indication	Antigen	Phase	NCT Number	Status	Study Start	Study Completion (Estimated)	Link
MS									
KYSA-7: A Phase 2, Open-Label, Randomized, Multicenter Study of KYV-101, an Autologous Fully Human Anti-CD19 Chimeric Antigen Receptor T-Cell (CD19 CAR T) Therapy, in Subjects With Refractory Primary and Secondary Progressive Multiple Sclerosis	n = 120	Primary and Secondary progressive MS	CD19	Phase 2	NCT06384976	Active, Not Recruiting	2024-09	2029-01	https://clinicaltrials.gov/study/NCT06384976
A Phase 1, Open-Label, Single Center Study of KYV-101, an Autologous Fully-Human Anti-CD19 Chimeric Antigen Receptor T-Cell (CD19 CAR T) Therapy in Subjects with Non-relapsing and Progressive Forms of Multiple Sclerosis	n = 12	Progressive MS	CD19	Phase 1	NCT06138132	Recruiting	2024-04-10	2027-06	https://clinicaltrials.gov/study/NCT06138132
Phase 1, Open-Label, Single Center Study of KYV-101, an Autologous Fully-Human Anti-CD19 Chimeric Antigen Receptor T Cell (CD19 CAR T) Therapy, in Participants With Treatment Refractory Progressive Multiple Sclerosis	n = 10	Progressive MS	CD19	Phase 1	NCT06451159	Active, Not Recruiting	2024-06	2027-06	https://clinicaltrials.gov/study/NCT06451159
NMOSD									
A Study on the Safety and Efficacy of Chimeric Antigen Receptor T Cells in the Treatment of Recurrent/Refractory Neuromyelitis Optica	n = 9	Relapsed and/ or Refractory NMOSD	CD19	Phase 1	NCT05828212	Recruiting	2023-04-30	2026-04-30	https://clinicaltrials.gov/study/NCT05828212
SPS									
KYSA-8: A Phase 2 Open-Label, Single-Arm, Multicenter Study of KYV 101, an Autologous Fully Human Anti-CD19 Chimeric Antigen Receptor T Cell (CD19 CAR T) Therapy, in Subjects With Treatment Refractory Stiff Person Syndrome	n = 25	Refractory SPS	CD19	Phase 2	NCT06588491	Recruiting	2024-09-25	2026-12	https://clinicaltrials.gov/study/NCT06588491
MG									
Autologous T-Cells Expressing A Chimeric Antigen Receptor Directed To B-Cell Maturation Antigen (BCMA) In Patients With Generalized Myasthenia Gravis (MG)	n = 30	Generalized MG	BCMA	Phase 2	NCT04146051	Active, Not Recruiting	2019-12-04	2026-03-31	https://clinicaltrials.gov/study/NCT04146051
KYSA-6: A Phase 2, Open-Label, Multicenter Study of KYV-101, an Autologous Fully Human Anti-CD19 Chimeric Antigen Receptor T-cell (CD19 CAR T) Therapy, in Subjects With Refractory Generalized Myasthenia Gravis	n = 20	Refractory generalized MG	CD19	Phase 2	NCT06193889	Recruiting	2024-08-28	2027-05	https://clinicaltrials.gov/study/NCT06193889
A Phase 1, Open-label, Safety and Dose-finding Study of Autologous Muscle-specific Tyrosine Kinase Chimeric Autoantibody Receptor T Cells (MuSK-CAART) in Subjects With Anti-MuSK-antibody-positive Myasthenia Gravis	n = 24	MuSK MG	MuSK	Phase 1	NCT05451212	Recruiting	2022-11-23	2028-10	https://clinicaltrials.gov/study/NCT05451212
MS, MG									
A Phase 1, Multicenter, Single-arm, Dose-escalation Study of CC-97540 (BMS-986353), CD19-Targeted NEX-T Chimeric Antigen Receptor (CAR) T Cells, Evaluating Safety and Tolerability in Participants With Autoimmune Neurological Diseases: Relapsing Forms of Multiple Sclerosis (RMS), Progressive Forms of Multiple Sclerosis (PMS), or Refractory Myasthenia Gravis (MG).	n = 120	Relapsing or Progressive MS or Refractory MG	CD19	Phase 1	NCT06220201	Recruiting	2024-03-28	2027-07-15	https://clinicaltrials.gov/study/NCT06220201
MS, NMSOD, MG									
An Exploratory Clinical Study of Cluster of Differentiation Antigen 20(CD20)/Anti-B-cell Maturation Antigen(BCMA) Chimeric Antigen Receptor Autologous T Cell Product (C-CAR168) in the Treatment of Autoimmune Diseases Refractory to Standard Therapy	n = 30	Refractory MS, NMSOD or MG	CD20 and BCMA	Phase 1	NCT06249438	Recruiting	2024-03-20	2040-03	https://clinicaltrials.gov/study/NCT06249438
MS, NMSOD, MG, CIPD									
An Exploratory Study on the Safety and Efficacy of Universal CAR-T Cells Targeting BCMA and CD19 in the Treatment of Refractory Autoimmune Diseases of the Nervous System	n = 25	Refractory MS, NMSOD, generalized MG or CIPD	CD19 and BCMA	Early Phase 1	NCT06485232	Not yet recruiting	2024-08-01	2027-12-31	https://clinicaltrials.gov/study/NCT06485232
B-cell/Antibody-mediated diseases (including MS, MG, NMOSD, MOGAD, AE, SPS, CIPD)									
An Open Label Clinical Trial to Evaluate the Safety and Efficacy of CT103A Cells for the Treatment of Relapsed/Refractory Antibody-associated Inflammatory Diseases of the Nervous System	n = 36	Relapsed/ refractory antibody-mediated inflammatory diseases of the nervous system	BCMA	Early Phase 1	NCT04561557	Recruiting	2020-09-22	2027-05-31	https://clinicaltrials.gov/study/NCT04561557
A Phase 1, Open-label Study to Evaluate the Safety and Clinical Activity of Azercabtagene Zapreleucel in Participants With B-cell Mediated Autoimmune Disorders	n = 32	B-cell mediated disorders	CD19	Phase 1	NCT06680037	Recruiting	2025-01-17	2029-01-01	https://clinicaltrials.gov/study/NCT06680037

### 3.1 Multiple Sclerosis (MS)

Multiple Sclerosis is a chronic autoimmune and neurodegenerative disease of the CNS characterized by initial myelin destruction and subsequent axonal pathology. The damage appears as lesions throughout the brain and spinal cord, interrupting key neuronal pathways leading to severe neurological impairment. The main autoantigens targeted by Th1/Th17, CD8+ T and B-cells are presumably myelin oligodendrocyte glycoprotein (MOG) and myelin basic protein (MBP). The therapeutic options rely mainly on immunomodulatory drugs that have therapeutic outcomes early in the disease progression when inflammatory demyelinating foci form (48).

Regarding CAR T-cells eliminating autoreactive cells, Yi et al., in 2022, using the MS-model of Experimental Autoimmune Encephalomyelitis (EAE) showed that peptide-MHCII CAR (pMHCII-CAR) T-cells prevented demyelination. The researchers engineered CAR T-cells but instead of expressing antigen-binding domain they expressed the complex MHCII-MOG_35-55_. As a result, the autoreactive CD4+ T-cells which bear high and low affinity TCRs that recognize this self-epitope, bound to the CAR T-cells and then became apoptotic, suggesting that affinity plays a role in the progression of EAE (49). Disease prevention was also revealed by Gupta et al., but this time they used anti-CD19 CAR T-cells for B-cell depletion. Clinical scores and lymphocyte infiltration were reduced in mice treated with CAR T-cells (50) contrasting the evidence of Mitsdoerffer et al. in 2021, who used the spontaneous opticospinal encephalomyelitis model, where mice develop spontaneous EAE in association with meningeal B-cell aggregates. Their research suggested that anti-CD19 CAR-T cells reduced the size of meningeal B-cell aggregates but exacerbated clinical disease probably due to the immunoregulatory role of these follicles (51).

In addition to the eliminating properties of CAR T-cells, Mekala and Geiger, back in 2005, provided early first evidence that genetically engineered Tregs could act therapeutically in MS, as supported by their results in an EAE model. They engineered CD4+ CD25+ Tregs using a chimeric receptor containing antigen-MHC and cytoplasmic signaling tail of TCR-zeta targeting MBP. Infusion of these cells resulted in symptoms alleviation and reduction of the pro-inflammatory milieu that promotes demyelination (52). Then, in 2012, Fransson et al., engineered anti-MOG CAR Tregs expressing a CAR for MOG and FoxP3, the gene responsible for development and function of Tregs. These CAR Tregs were tested, first *in vitro* and then *in vivo* and showed not only dampening disease severity but also prolonged effects of CAR Tregs. Disease-free mice, when re-administered the EAE induction protocol, remained disease-free (53). Furthermore, in 2020, De Paula Pohl et al., demonstrated that human CD4^+^CD25^hi^CD127^low^ Tregs expressing chimeric receptors targeting either MBP or MOG when transfused in EAE mice, promoted tolerance in autoreactive effector T-cells and hindered disease progression (54).

There are several clinical trials of CAR T-cells in MS patients. In 2024, FDA approved two phase 1 clinical trials of CD19-CAR T-cell therapy named KYV-101. The first one refers to patients with non-relapsing and progressive forms of MS (NCT06138132) (55) while the second includes only patients with the progressive forms (NCT06451159) (56). In the same year, Fischbach et al., published the first evidence of efficacy and safety of treatment with KYV-101 in two patients with progressive MS. Both patients manifested low-grade Cytokine Release Syndrome (CRS), inflammatory relapses did not occur, and CAR T-cells expanded within the cerebrospinal fluid (CSF) highlighting their ability in penetrating CNS (57). This is consistent with recent *in vitro* studies which suggest that autoimmune-patient-derived CD19 CAR T-cells possess efficient cytotoxicity without producing high levels of cytokines that could be harmful (58). Additionally, there is another clinical trial for KYV-101, named KYSA-7. It is a phase 2 study and includes patients with Refractory Primary and Secondary Progressive MS (NCT06384976) (59).

### 3.2 Myasthenia Gravis (MG)

Myasthenia gravis is the most common autoimmune disease affecting the neuromuscular junction (NMJ) of the skeletal muscles leading to muscle weakness especially of the eyes, throat, and extremities. The auto-Abs secreted by plasma cells target cell surface proteins of the post-synaptic terminal of muscle cells and thus neurotransmission is hindered. There are several types of MG linked to a specific auto-Ab. Nicotinic acetylcholine receptors (n-AChRs), muscle-specific kinase (MuSK), and lipoprotein-related protein 4 (LPR4) are the main targets of humoral immunity. 12% of patients with MG have thymoma which is responsible for the auto-Abs production. Monoclonal antibodies for B-cell depletion and complement pathway inhibition along with corticosteroids are the current therapeutic options (60).

The first findings from a prospective, multicenter, open-label, phase 1b/2a clinical trial anti-BCMA rCAR T-cells (NCT04146051) (61) exhibited promising therapeutic outcomes in adult patients with generalized MG. In this study, Granit et al., transduced T-cells with RNA (rCAR-T), rather than DNA similar to mRNA vaccines for SARS-CoV-2, having in mind a better safety profile. B-cell Activating Factor (BAFF) and A Proliferation-Inducing Ligand (APRIL) levels, which are prognostic markers of MG, were found reduced suggesting that B-cells were eliminated as both markers correlate with survival and proliferation of B-cells. Also, hypogammaglobulinemia or susceptibility to infection was not observed. Treatment was well-tolerated by patients who demonstrated substantial numerical decrease in MG scales compared to other MG clinical trials, paving the way for further investigation of the potential of cell therapy in MG (62).

Moreover, in late 2023, Haghikia et al., reported the first case of a female patient treated with anti-CD19 CAR T-cells (KYV-101). The woman suffered from a treatment-resistant MG and over the first 2 months after cell infusion her clinical condition improved dramatically. She did not experience side effects like CRS or cytopenia but only mild transaminitis that did not necessitated therapy. Also, anti-AchR antibodies were reduced by 70% due to successful CD19+ B-cells elimination. This evidence suggests that KYV-101 is a desirable alternative “in the battle” against MG (63) and the ongoing phase 1/2 clinical trial (KYSA-6) in patients with refractory MG will provide more insights (NCT06193889) (64).

In parallel, the promising preclinical data presented by Oh et al. (65), paved the way for an ongoing open-label, safety and dose-finding phase 1 clinical trial of MuSK-CAAR T-cells in combination with cyclophosphamide or cyclophosphamide and fludarabine (both immunosuppressants) in patients with active, anti-MUSK-Abs+ MG (NCT05451212) (66). Researchers developed MuSK-CAAR T-cells against B-cells. The CAAR T-cells expressed epitopes of the autoantigen MuSK which autoreactive B-cells bind and become apoptotic. They managed to selectively eliminate MuSK-specific B-cells in an experimental autoimmune MG mouse model without causing hypoglobulinemia or depletion of other B-cell populations (65).

Lastly, there is an ongoing phase 1 clinical trial of anti-CD19 CAR T-cells combined with fludarabine and cyclophosphamide for patients with relapsing or progressive MS or refractory MG (NCT06220201) (67).

### 3.3 Neuromyelitis optica spectrum disorder (NMOSD)

Neuromyelitis optica spectrum disorder is an inflammatory demyelinating disorder of CNS. IgG antibodies against aquaporin-4 (AQP4-IgG) that often mobilize complement, is the main serological finding in the majority of NMOSD patients. Aquaporin-4 is a water channel found on the foot processes of astrocytes and is highly concentrated in certain parts of the CNS, such as the optic nerve, spinal cord, and brainstem, which are severely affected by the disease. Currently, AQP4-IgG+ NMOSD is treated with mAbs targeting complement protein C5 or B-cells or IL-6 receptor, while in seronegative NMOSD immunosuppressive agents like azathioprine are employed (68).

Qin et al., reported the safety profile and the promising therapeutic outcomes of the anti-BCMA CAR T-cell therapy named CT103A in patients with relapsed or refractory AQP4-IgG+ NMOSD. Almost all patients did not have relapses for approximately 6 months, possibly due to the decreased AQP4-IgG levels in the serum, while disability was also improved. Neurotoxicity did not occur, and CRS scored grade 1–2. All patients though exhibited hematotoxicity and infections even in grade 3, with two patients over 60 years, requiring hospitalization (69). Moreover, there is an ongoing phase 1 study about the safety and efficacy of CD19 CAR-T therapy for patients with relapsed or refractory NMOSD (NCT05828212) (70).

Currently, there is a phase 1 clinical trial of CD20/BCMA-directed CAR-T cells for patients with refractory MS, MG, NMOSD (NCT06249438) (71).

### 3.4 MOG (Myelin oligodendrocyte glycoprotein) antibody-associated disease (MOGAD)

Myelin oligodendrocyte glycoprotein (MOG) is a protein expressed exclusively on the surface of oligodendrocytes in the CNS contributing to complete myelin formation. Although MOG may also be an autoantigen in MS, as shown mainly in animal models (72), the phenotype of the MOG-IgG+ inflammatory disease is distinctly different from that of MS. It is the most recently defined demyelinating disease of the CNS and while very rare, it has a differential clinical course and alternative treatment. The clinical presentations range from isolated optic neuritis or myelitis to multifocal CNS demyelination. In children, Acute Disseminated Encephalomyelitis (ADEM) is the most frequent presentation. Currently, there are no FDA-approved B cell-depleting therapies for MOGAD, and corticosteroids serve as the first option. Also, azathioprine, mycophenolate mofetil, and rituximab alleviate relapses (73).

Cabrera-Maqueda et al. recently demonstrated a case-report of an 18-year-old male patient with refractory MOGAD treated with CD19-directed CAR T-cells (ARI-0001 cells). CD19+ cells were quickly eliminated, MOG-IgG was tested null and both memory B-cells and plasmablasts did not appear for more than 6 months, suggesting that CAR T-cells can be successful as a therapeutic intervention for MOGAD (74).

### 3.5 Autoimmune encephalitis (AE)

Autoimmune encephalitis refers to a group of neurological conditions arising from auto-reactive cells’ assault in the brain causing inflammation which leads gradually to severe functional impairments of the CNS. AE is frequently associated with underlying malignancy. There is a plethora of auto-Abs that target autoantigens in neuronal membranes and in synapses. Each Ab correlates with a distinct type of encephalitis. For example, anti-NMDA (Anti-N-methyl-D-aspartate) receptor (NMDAR) auto-Abs give rise to anti-NMDAR encephalitis, the most common type. However, there are seronegative forms that exhibit absence of detectable auto-Abs in the serum or CSF (75).

Anti-NMDAR encephalitis is characterized by auto-Abs mainly of IgG1 and IgG3 subclasses directed against the NR1 subunit of the NMDA receptor in the CNS leading in psychosis, seizures, cognitive decline and motor symptoms. The NMDAR is a glutamate receptor and Ca^2+^ ion channel located in the cell surface of neurons and is part of excitatory CNS synapses. In some patients, ovarian teratoma is the source of the antigen which triggers the production of the auto-Abs (76).

Reincke et al., in 2023, engineered NMDAR-specific chimeric auto-Ab receptor (NMDAR-CAAR) T cells to eliminate B-cells which produce anti-NMDAR Abs. CAAR T-cells had an NMDAR autoantigen, instead of scFv, which is targeted by autoreactive B-cells through their BCRs. After the interaction with the CAAR T-cells, the latter release effector molecules that induce lysis of the B-cells. Both the *in vitro* and *in vivo* assays were promising highlighting their possible therapeutic outcomes in patients. CAAR T-cells were effective in eliminating their target *in vitro* and decreased the auto-Abs in serum and brain with no side effects in mice (77). Clinical trials are needed to ensure the safety and efficacy in patients.

### 3.6 Stiff-person syndrome (SPS) spectrum disorders

Stiff-person syndrome spectrum disorders comprise a group of rare autoimmune neurological diseases with high heterogeneity but mainly characterized by painful muscle spasms in the lower extremity and less often in upper ones. Most patients are seropositive in Abs against glutamic acid decarboxylase 65 (GAD65) enzyme (anti-GAD65 Abs) giving rise to classical SPS. GAD65 is present in GABAergic pre-synaptic neurons which produce the inhibitory neurotransmitter GABA (γ-aminobutyric acid) through decarboxylation of glutamate. In SPS, GAD65 is mistakenly recognized as an antigen and processed by antigen-presenting cells which orchestrate the production of auto-Abs against it through the activation of T and B cells. There are also patients who have Abs against amphiphysin, a pre-synaptic vesicle protein involved in endocytosis at synapses. These Abs associate with an underlying paraneoplastic syndrome that gives rise to SPS symptoms (paraneoplastic SPS). Benzodiazepines and immunosuppressants are the main therapeutic options (78).

Recently though, a successful application of anti-CD19 CAR T-cell therapy (KYV-101) in a female patient with refractory SPS has been reported, as the reduction of anti-GAD65 titers led to stiffness reduction, too. Benzodiazepines’ use was limited to 60% and there was no need for immunosuppressants’ administration after CAR T-cell infusion. Faissner et al., speculated that the symptoms were alleviated because CAR T-cells were present in CSF, as also observed in MS patients (79). Recently, the FDA approved an open-label phase 2 clinical trial of KYV-101 for patients with treatment refractory SPS named KYSA-8 (NCT06588491) (80).

### 3.7 Chronic inflammatory demyelinating polyneuropathy (CIDP)

Chronic inflammatory demyelinating polyneuropathy is an immune-mediated neuropathy affecting PNS and nerve roots. The typical form of the disease manifests symmetric, motor weakness and vibration and position-sense impairment. Most cases of CIDP are idiopathic, although preceding infections are strongly correlated with the disease onset. Both humoral and cell-mediated immunity are involved and 10% of patients with CIDP are seropositive for IgG4 and IgM auto-Abs mainly against neurofascin (NF) isoforms (NF155, NF140, NF186) or contactin-1 (CNTN1); all of them are proteins in nodal and paranodal Ranvier regions. Corticosteroids, Intravenous Immune Globulin (IVIG), and plasma exchange serve as first-line treatment options (81).

Zhang et al., in 2024, reported the case of a male-patient with refractory CIPD who received bispecific CD19-BCMA CAR T-cells. Despite the grade 1 CRS, CD19+ B-cells reached 0 levels 10 days after the CAR T-cell infusion. Also, the fact that he did not have disease relapses in the 1-year follow-up and did not receive immunosuppressants all this time-period, is impressive (82).

Currently, there is an early phase 1 clinical trial of CD19- and BCMA CAR T-cells in patients with refractory MS, NMSOD, generalized MG or CIPD (NCT06485232) (83). In parallel, there are two more phase 1 clinical trials (NCT04561557, NCT06680037) of CAR T-cells targeting BCMA and CD19, respectively, in patients with B-cell/Antibody-driven autoimmune disorders, including all the above-mentioned ones (MS, MG, NMOSD, MOGAD, AE, SPS, CIPD) (84, 85). These trials are expected to provide insights into the efficacy, toxicities and proper dosage of CAR T-cells.

## 4 Limitations of CAR T-cell therapy

### 4.1 Adverse effects, safety, persistence and practical issues

CAR T-cells play a critical role in targeting and eliminating tumor cells through proliferation, a process that necessitates the production of pro-inflammatory cytokines. However, excessive cytokine production can lead to systemic inflammatory responses, such as cytokine release syndrome (CRS) and immune effector cell-associated neurotoxicity syndrome (ICANS) (29, 40, 86, 87). CRS often includes macrophage activation syndrome (MAS), contributing to a life-threatening cytokine storm syndrome (CSS), known as hemophagocytic lymphohistiocytosis (HLH) (86, 87). ICANS, on the other hand, involves increased cytokine levels in the cerebrospinal fluid (CSF), compromising the blood–brain barrier (BBB) and endangering the brain parenchyma. Both CRS and ICANS can present with symptoms ranging from mild (such as headache, diarrhea, speech difficulties, and seizures) to severe (including heart, respiratory, or multiorgan system failure, cerebral edema, and coma), classified from grade 1 to 5 respectively. CRS can also cause hematotoxicity, with conditions like anemia, neutropenia, and thrombocytopenia commonly occurring after CAR T-cell infusion. Therefore, administration of corticosteroids and tocilizumab, an IL-6 receptor monoclonal antibody, is crucial in managing these conditions due to IL-6’s significant role in their onset (29, 40, 86, 87).

In the context of hematological malignancies, CRS incidence is anticipated to be severe owing to the abundance of tumor cell targets compared to autoimmune neurological diseases. A lower number of targets generally result in less CAR T-cell expansion and thus a lower grade of CRS (86, 87). This finding aligns with a meta-analysis by Anagnostou et al., which reviewed 35 clinical trials of anti-CD19 CAR T-cell therapy for patients with relapsed/refractory acute lymphoblastic leukemia (ALL), showing grade 3 or higher CRS and ICANS in 26% and 12% of cases, respectively (88). Although CAR T-cell therapy for autoimmune neurological ailments is still in its early deployment stages, there have been no reports of ICANS, and observed CRS is typically only grade 1–2 (57, 62, 63, 77, 79, 82). One clinical trial reported grade 3 CRS, hematotoxicity, and infections in anti-BCMA CAR T-cell-treated NMOSD patients, with two requiring hospitalization. However, these individuals were over 60 and chronic immunosuppressant users, compromising their immune systems, so these findings should be cautiously considered (69).

Several agents, including monoclonal antibodies (mAbs), corticosteroids, and calcineurin inhibitors, can effectively deplete B or T-cell autoreactive populations or inhibit pro-inflammatory cytokines, alleviating symptoms (12, 89). However, they do not offer the curative potential that CAR T-cells do (20, 29, 57, 62, 63, 69, 74, 77, 79, 82). For instance, B-cell targeting mAbs have a limited half-life, necessitating repeated doses to maintain the depletion of pathological cells (12, 89, 90). CAR T-cell therapies promise to eradicate autoreactive cells with a single infusion, providing extended periods without symptoms or disease recurrence due to their *in vivo* expansion and persistence (57, 62, 63, 69, 74, 77, 79, 82). Moreover, mAbs cannot reach autoreactive cells in tissues or ectopic germinal centers, which is crucial for autoimmune neurological diseases, where pathology may reside within the nervous system or meninges (12, 91). CAR T-cells can traverse the BBB to eliminate autoreactive cell populations responsible for the disease (57, 69, 74, 77, 79). Nonetheless, concerning side effects, the heightened risk of infection or osteoporosis from chronic mAb or corticosteroid use (12, 14, 92) is often preferable to potential life-threatening CAR T-cell therapy complications, like CRS (86, 87). mAbs are generally well-tolerated and do not induce organ toxicity (12, 14, 89, 90).

Additionally, the mechanism of action of mAbs and CAR T-cells differ significantly. For example, Rituximab, an anti-CD20 antibody, stimulates B cell depletion primarily through antibody-dependent cellular cytotoxicity (ADCC) via NK cells and complement-dependent cytotoxicity (CDC). In a phase 2 clinical trial in relapsing-remitting MS (RRMS), rituximab led to a 91% reduction in demyelinating lesions (93). However, it failed in phase 3 trials for systemic lupus erythematosus (SLE), involving patients with moderate-to-severe non-renal SLE (94). This limited efficacy of Rituximab may be due to the heterogeneity of autoreactive B cell clones in SLE patients as well as the absence of CD20 on long-lived plasma cells. Although combinations of mAbs targeting different B cell markers have attempted to overcome these limitations, such approaches have been associated with exacerbation of symptoms and increased infection risk (12). Remarkably, in a small cohort of five patients with refractory SLE treated with anti-CD19 CAR T cells, all achieved clinical remission within three months (94). Maybe this can be attributed to the fact that CAR T cells recognize a surface target (CD19) which is present in both long and short-lived plasma cells and directly mediate their apoptosis via cytokine release and lytic enzymes.

Another emerging issue with CAR T-cells is their unknown long-term immunological impact. Unlike drugs that can be modified or discontinued in case of side effects, CAR T-cells are “living” therapies, so the host’s immune system might recognize CAR components as foreign, attempting to eliminate them (23, 29, 87). Currently, there is no data on their prolonged effects on patient physiology. Additionally, CAR T-cells are an expensive and complex “tailor-made” treatment, requiring time and being limited to highly specialized centers worldwide, which restricts patient access (95). Particularly, time constraints disadvantage patients with severe relapses needing immediate care. Immunosuppressants efficiently control inflammation promptly (12, 89). As of now, there is no evidence of CAR T-cell efficacy and safety in relapsing autoimmune neurological diseases like Relapsing-Remitting MS, as most trials focus on treatment-resistant or progressive forms with established organ damage (55, 56, 59, 64, 67, 70, 71, 83–85).

Lastly, identifying specific biomarkers indicative of patient response is essential. For instance, in progressive MS, inflammation is accompanied by neurodegeneration, making monitoring challenging as MRI scans don’t reveal disease progression or treatment response. Screening for auto-antibodies involved in a disease, when present, is insufficient (96). Further research is needed to determine the effectiveness of cell therapy in progressive autoimmune neurological disease phases.

### 4.2 Antigen selection/escape, on-target off-tumor effects and resistance

CAR T-cell therapy, unlike some immunomodulatory drugs, impacts only the targeted cell population rather than the whole immune system. Targeting specific antigens on specific cells is accompanied by less immunosuppression and thus lower infection risk. Therefore, antigen selection ensures CAR efficacy and safety as “on-target off-tumor” toxicity is undesirable. CAR T-cells must not cross-react with antigens that are also expressed on normal cells apart from tumor or autoreactive ones (97–99). There are ongoing attempts to find antigens that are highly distributed in tumor cells and do not appear or appear in low numbers in normal cells. However, even if we identify the “perfect antigen”, tumors may exhibit antigen escape via downregulation or mutation of this antigen. CAR-T cells may not recognize the altered or mutated version of the antigen, thus leading to CAR-T cell resistance (87, 97–99). Does this also apply to autoimmune diseases? In autoimmunity, according to the neoantigen (100) and epitope spreading hypothesis (101) there is an amplification in the number of autoantigens due to the formation of new or revelation of previously sequestered antigens, respectively (100, 101). Bearing these hypotheses in mind, maybe, CAR T-cells resistance occurs but further clinical trials will elucidate it. However, the CAAR-based approach seems to be unsuccessful against the plasma cells and especially the long-lived ones, since they decrease their BCR expression during their differentiation from precursors (102).

Resistance can be also established by CAR T-cell exhaustion when CAR T-cells exhibit less proliferation, cytotoxicity, and cytokine secretion. In cancer, this is a result of the profound antigen-burden of tumor cells and the immunosuppressive tumor microenvironment which inhibits CAR T-cells (97–99, 103). We expect less CAR T-cell exhaustion at the onset of an autoimmune disease as it does not possess such a range of autoantigens like cancer (99, 100). However, if we consider that inflammation plays a role in T-cell exhaustion (104) and the pro-inflammatory milieu of patients with autoimmunity is heightened, sometimes even if it is treated (105, 106), maybe we cannot avoid CAR T-cell exhaustion. Chronic autoimmune disorders may require long-lived effector CAR T-cells (23, 27, 94), but this is impossible (100, 101, 103). Until now, there was an AQP4-IgG+ NMOSD patient treated with anti-BCMA CAR T-cell therapy who had a possible attack of decreased visual acuity in the left eye 14 months after infusion (69). One can attribute it to CAR T-cell exhaustion, but further investigation is needed to confirm this assumption.

## 5 Discussion

The potential of CAR T-cell therapy in the treatment or even cure of autoimmune diseases is currently being heavily investigated expanding the knowledge gained from hematological malignancies treated with this innovative modality. Particularly, autoimmune neurological diseases need novel therapeutic strategies as a minority of patients do not respond to immunomodulatory drugs and mAbs fall short in infiltrating tissues like brain parenchyma and depleting the autoreactive cells as in case of Rituximab and SLE patients. CAR T-cells, although the clinical evidence is still limited, may offer long-lasting benefits with longer treatment-free remissions and with manageable complications. The fact that CAR T-cells migrate within the CNS crossing the BBB and eliminate autoreactive cells with high specificity is notable. However, there is an urgent need for more pre-clinical and clinical data as their precise engineering ensures a better safety profile and efficacy. Long-term clinical trials with larger numbers of subjects will track the optimal CAR T-cell dosage and minimize adverse effects like CRS. Moreover, clinical trials should include not only patients with refractory autoimmune neurological diseases but also those with the inflammatory/autoimmune activity of the disease at its peak, before sustained damage occurs. Furthermore, apart from the conventional CAR T-cells, CAR Tregs are necessary to come in the limelight as clinical trials for malignancies report their superior safety profile. In parallel, there are ongoing efforts to engineer novel CAR structures which will target multiple antigens, produce immunomodulatory cytokines or even “suicide” of CAR T-cells after they serve their role to mitigate the toxicities. It remains to be seen if these breakthroughs in the CAR designs will become broadly available and effective therapies for cancer and autoimmune diseases.

Nevertheless, the cost-benefit equilibrium should be assessed carefully. Life-threatening complications are not acceptable in autoimmune diseases since most of them reduce the quality of life of patients compared to the inevitable demise -without proper treatment- caused by cancer. The prospect of cure though is tempting and even if possible with unwanted side effects, patients may still desire to participate in clinical trials. Recruitment of the appropriate patients and keeping in mind that benefits must outweigh detriments, is essential.
